# A canine-derived chimeric antibody with high neutralizing activity against canine parvovirus-2

**DOI:** 10.1186/s13568-022-01416-8

**Published:** 2022-06-15

**Authors:** Lixuan Zhou, Hongchao Wu, Mengmeng Du, Huanhuan Song, Ningning Huo, Xiao Chen, Xiaorui Su, Weiguo Li, Lulu Wang, Jie Wang, Baicheng Huang, Feifei Tan, Kegong Tian

**Affiliations:** 1 College of Veterinary Medicine, Henan Agricultural University, Zhengzhou, China; 2 National Research Center for Veterinary Medicine, Luoyang, China; 3 Luoyang Huizhong Biotech Co., Ltd., Luoyang, China

**Keywords:** Canine parvovirus-2, Chimeric antibody, CHO cell expression system, Therapeutic effect

## Abstract

Canine parvovirus-2 (CPV-2) infection causes serious multisystemic disease in dogs and many animal species worldwide. Previously, a monoclonal antibody (MAb) of CPV-2, 10H4, showed high neutralizing activity and therapeutic effect against CPV-2 in dogs. However, the application of mouse MAb is limited in other animals due to immune rejection. Here, the variable regions of the heavy and light chains of 10H4 were cloned and ligated with constant canine antibody regions to produce a canine-derived chimeric MAb 11D9, in a CHO-S cell expression system. The cell supernatant of the CHO cell line 11D9 exhibited a HI titer of 1:2560 against all the variants of CPV-2 (new CPV-2a, new CPV-2b, and CPV-2c), and had the same average neutralization titer as the new CPV-2a (1:11,046.5) and new CPV-2b (1:11,046.5) variants, which was slightly higher than that of CPV-2c variants (1:10,615.7). In animal experiment, the treatment of chimeric MAb 11D9 had a high therapeutic effect in beagles infected with the new CPV-2a. Overall, the canine-derived chimeric MAb 11D9 produced by CHO-S cells showed a high HI and neutralization titer against CPV-2 and the therapeutic effects against the new CPV-2a in beagles, providing potential for the prevention or treatment of CPV-2 infections in dogs.

## Key points

- A canine-derived chimeric MAb 11D9 was produced by CHO cell lines.
- The MAb 11D9 exhibited high HI and neutralization titers against new CPV-2 variants.
- The MAb 11D9 had a high therapeutic effect in beagles infected with the new CPV-2a variant.

## Introduction

Canine parvovirus type 2 (CPV-2) can cause a highly fatal and contagious systemic disease in dogs and many animal species worldwide (Miranda and Thompson 2016; Organtini et al. 2015). CPV-1 infection, or minute virus of canines, has no clinical signs, which is distinct from CPV-2 infection (Lamm and Rezabek 2008; Ohshima et al. 2004). A local endemic trend of CPV-2 infections with different morbidities and mortalities has been observed in China (Zhao et al. 2013). CPV-2 is prone to mutation, identified as the mutated strains new CPV-2a, new CPV-2b, and CPV-2c. Among those strains, the new CPV-2a, and new CPV-2b are spreading worldwide (Chen et al. 2021). The dominant strains currently circulating in China are new CPV-2a, new CPV-2b, and CPV-2c (Casertano et al. 2020). Phylogenetically, the gene sequence similarity of new CPV-2a and new CPV-2b is higher than that of CPV-2c (Qi et al. 2020).

Passive immunization with monoclonal antibody (MAb) is beneficial in reducing the severity of CPV-2 infection. MAb is currently used in combination with symptomatic treatments for treating CPV-2 infections; compared to symptomatic treatment alone, this method results in higher survival rates (Mazzaferro 2020).

Previously, MAb 10H4, which neutralizes new CPV-2a, was produced against canine parvovirus. IgG2a/Kappa is the classification of the heavy and light chains of the mouse monoclonal antibody (MAb) 10H4 (Hao et al. 2017). However, using murine MAb in canine treatment, particularly with repeated administration, may be restricted by their immune rejection, which can lead to serum sickness and the ‘‘foreign’’ antibodies being rapidly cleared from their circulation(Guirakhoo et al. 2001). To avoid the immune rejection caused by MAb, the humanized antibodies to treat human diseases have been developed (Casertano et al. 2020). However, no such technology has been widely used in animal disease treatments. Here, a canine-derived chimeric MAb (expressed by the CHO cell line) was constructed based on MAb 10H4.

## Materials and methods

### Animals

Beagles (n = 10) used in this study were tested antibody negative for CPV-2. All the animal samples were collected according to the protocol approved by the Animal Care and Ethics Committee of the National Research Center for Veterinary Medicine.

### MAb of CPV-2 and virus

Murine MAb 10H4 (CPV-2 VP2 specific) was obtained from the National Research Center for Veterinary Medicine, which was produced by the immunization of mice with partially purified new CPV-2a virus (strain CVCC AV298). The host cell lines used in the expression of recombinant antibodies included the ExpiCHO-S (Thermo Scientific, USA) and CHO-S cell lines (Thermo Scientific, USA). The virus of new CPV-2a, new CPV-2b, and CPV-2c were provided by the National Research Center for Veterinary Medicine.

### Cloning and sequencing of the variable heavy (VH) and variable light (VL) regions

The variable gene sequences of the MAb clone were amplified. Briefly, 10^6^ hybridoma A011 cells (MAb 10H4, deposited by the National Research Center for Veterinary Medicine) were collected by centrifugation. Total RNA was extracted using RNeasy Mini Kit (QIAGEN, USA) according to the manufacturer’s protocol. After reverse transcription and PCR amplification, the PCR products were identified by agarose gel electrophoresis and purified using a commercial kit (Tiangen, China). The DNA fragments were cloned into the Blunt vector (TransGen Biotech, China) and sequenced individually (GENEWIZ, China).

### Construction of the chimeric antibody expression vector

**Fig. 1:**
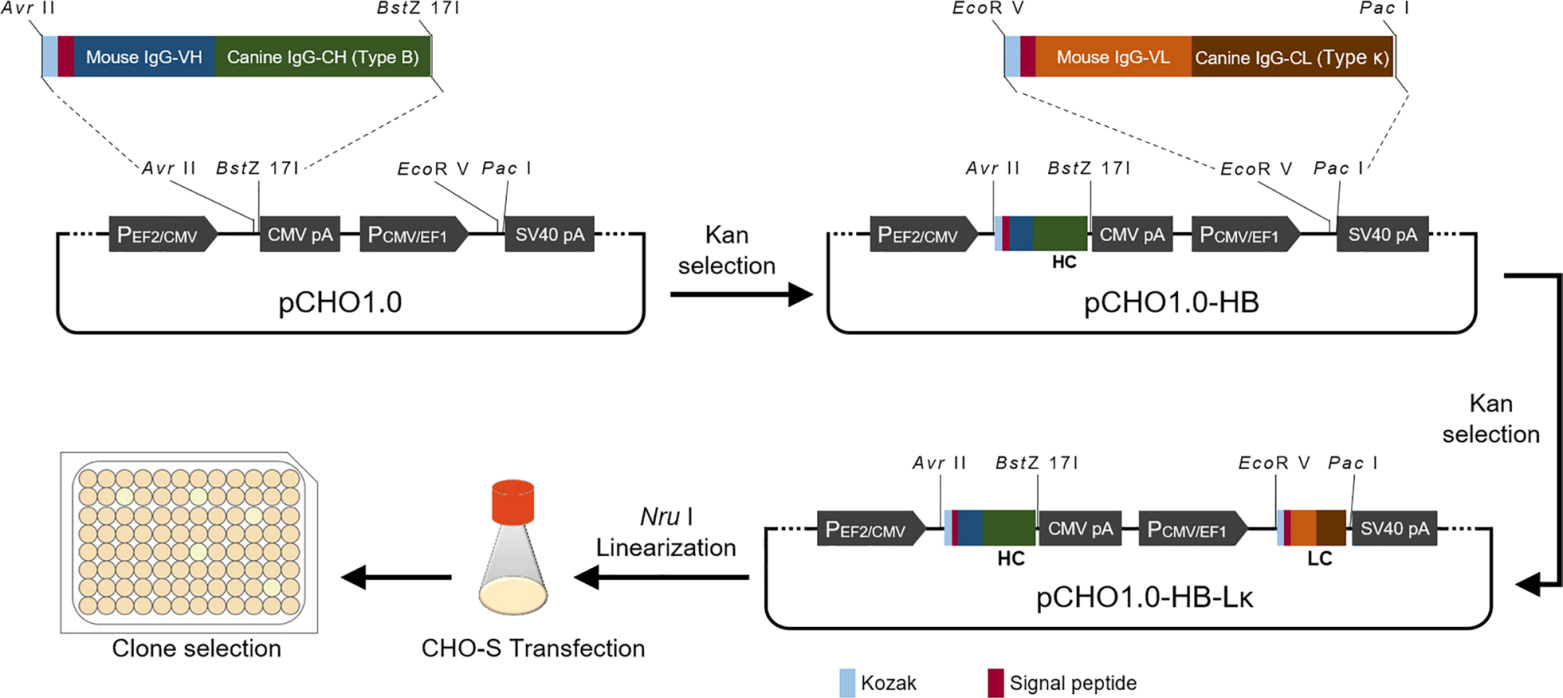
Schematic design of the chimeric antibody construction

The canine IgG-B (Genbank accession number: AF354265) constant region sequence was linked with the murine heavy chain variable region sequence. The light chain variable region sequence was combined with the canine IgL-κ (IMGT accession number: E02906) constant region sequence. These complete sequences were all supplemented with a signal peptide and KOZAK sequences. Two restriction sites (*Avr* II and *BstZ* 17I) were added to the chimeric antibody heavy chain, and 2 restriction sites (*Eco*R V and *Pac* I) were added to the chimeric antibody light chain. Both chimeric antibody heavy chain and light chain genes were optimized and synthesized (GENEWIZ, China). pCHO1.0 can achieve the dual expression of two proteins by inserting two gene sequences into the two independent insertion sites (Fig. 1). The newly generated pCHO1.0-HB-Lκ construct was transformed into *Escherichia coli* Top10 cells, and the DNA sequence of the chimeric antibody was confirmed by sequencing.

### Transient expression of canine-derived chimeric MAb in ExpiCHO-S

pCHO1.0-HB-Lκ was transferred into ExpiCHO-S cells using the ExpiFectamine CHO Transfection Kit (Thermo Scientific, USA). All experimental operations were performed according to the manufacturer’s protocol. An aliquot of spent growth medium was collected after 10 days and then centrifuged to obtain the cell supernatant.

### Chimeric antibody identification by SDS-PAGE and Western blot

Self-cast 12% SDS–acrylamide gels were used for the protein gel electrophoresis. Semidry blotting was performed according to standard blotting protocols using nitrocellulose membranes. Blocking was carried out by a phosphate-buffered saline buffer (PBS: 50 mmol/L, 150 mmol/L NaCl; pH 7.5) with 0.05% Tween 20 (PBST) + 5% Skim Milk incubation for 1 h at room temperature. For detecting the chimeric antibody, HRP-labelled goat anti-canine IgG (H + L) secondary antibody (Thermo Scientific) was used at a 1:1000 dilution for 1 h. After washing three times with PBST for 5 min, the binding of the HRP-conjugated antibodies was detected by incubation with DAB KIT (Tiangen, China).

### Hemagglutination inhibition (HI) test

PBS (pH 7.2) was used for the HI buffer, and cell supernatant was diluted to 1:10 (as the initial concentration for detection). Subsequently, twofold serial dilutions of the cell supernatant (25 μl) were mixed with viruses (8 hemagglutination units, HAU/25 μl) and incubated at 37 °C for 30 min. At the same time, new CPV-2a antigen and red blood cell control wells were tested. Then, 50 μl of a HI buffer containing 1% porcine erythrocytes was added, and the mixture was shaken and maintained at 4 °C for 2 h. The HI titer was expressed as the reciprocal of the highest dilution that completely inhibited viral hemagglutination.

### Neutralization test

Two-fold serial dilutions of cell supernatant (220 μl) were mixed with viruses (100 TCID_50_/220 μl) in a 5% carbon dioxide and 95% atmosphere at 37 °C for 90 min. Then, the mixture (100 μl, with 4 replicates) was added to 96-well plates in which feline kidney F81 cells (2 × 10^4^ cells/100 μl) had been previously added. The 96-well plates were placed in 5% carbon dioxide and 95% atmosphere at 37 °C. After 5 days, the medium was discarded, and the cells were fixed in the wells of 96-well plates with cold acetone for 30 min at 4 °C. After being washed three times with PBS, the cells were incubated with the mouse MAb 10H4 (1:1000 diluted) at 37 °C for 50 min, and then a 1:200 dilution of FITC-conjugated goat anti-mouse (Sigma, 100 μl/well) was added and incubated at 37 °C for 50 min. After being washed three more times, PBS (50 μl/well) was added, and the cells were observed under fluorescence microscopy.

### Selection of stable cell lines for mouse-canine chimeric antibody production

According to the manufacturer’s protocol, the plasmid was linearized by *Nru* I and then transferred into CHO-S cells using FreeStyle MAX reagent (Thermo Fisher). Complete CD FortiCHO medium containing a combination of puromycin and methotrexate was used to select stable transfectants. To identify whether the selected cells could express protein, the cell supernatants were identified by Western blot. Cell pools expressing mouse-canine chimeric antibodies were selected for cloning by limiting dilution. In brief, the cells in the cell pool were diluted to 2.5 cells per milliliter, and the cells were added to a 96-well plate, that is, 0.5 cells per well. When the cell confluence was 10 ~ 90%, the monoclonal cell supernatant was collected, and a dot blot (HRP-labelled goat anti-canine IgG for screening) was used for identification. The monoclonal cells expressing chimeric antibodies were transferred to 24-well plates. After the cells were grown in a 24-well plate for 2–5 days and then subjected to a second dot blot screen, the cells with high chimeric antibody expression were transferred to a 6-well plate for culture. After the cells reached a sufficient density, they were transferred to a cell shake flask fed culture and subjected to SDS-PAGE, HI, and neutralization tests (against new CPV-2a) to identify cell lines with a high chimeric antibody expression and neutralizing activity. We performed the 2nd round selection (limiting dilution cloning) for cell line construction.

### Evaluation of the biological characteristics of canine-derived chimeric MAb

The cell supernatant of the canine-derived chimeric MAb (expressed by a CHO cell line) was identified by reducing and non-reducing SDS-PAGE. The following three existing CPV-2 variant strains, new CPV-2a, new CPV-2b, and CPV-2c, in our laboratory were used for HI and neutralization tests.

### Therapeutic efficacy of the canine-derived chimeric MAb in beagles

**Fig. 2:**
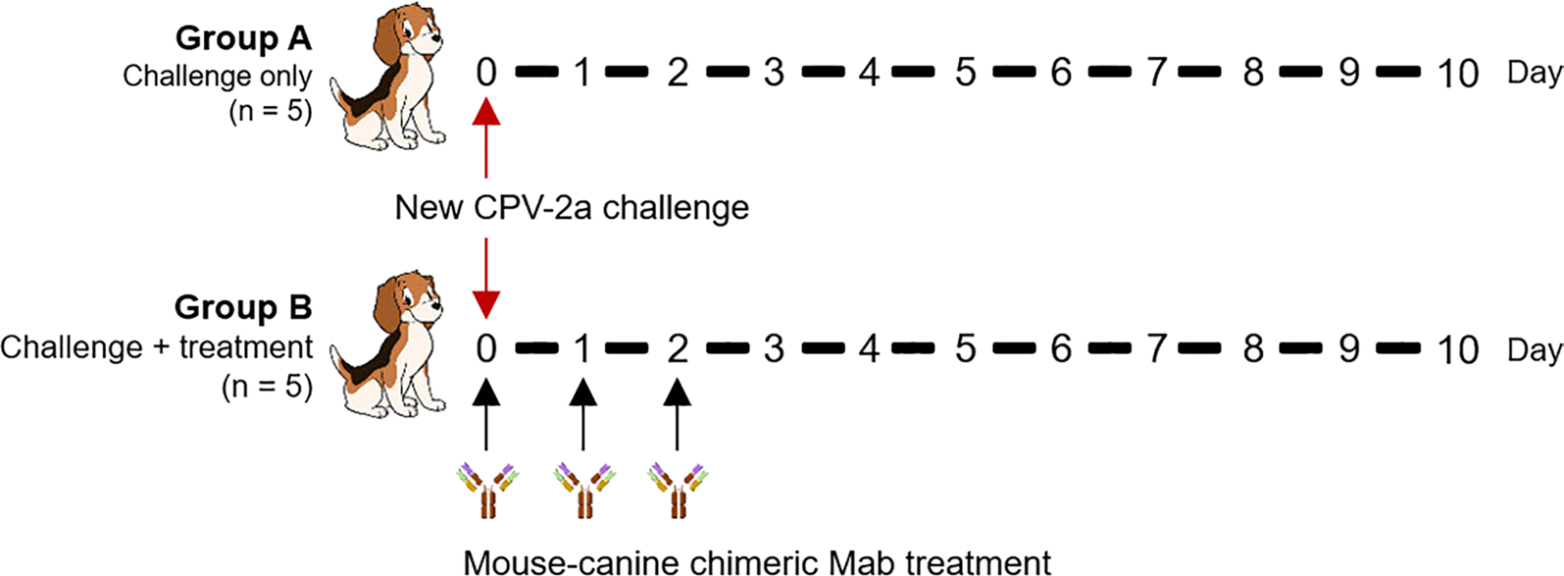
The design of the therapeutic efficacy study of 11D9 in beagles

Ten beagle dogs (100-day-old) that were seronegative for CPV-2 were randomly divided into 2 groups. A single dose of the new CPV-2a virus (10^6.50^ FAID_50_/mL, 4.0 mL) was injected subcutaneously into each group (n = 5). Six-hour after the challenge, group A (challenge group) was injected with PBS, and group B (treatment group) received a three-day intramuscular injection with a single dose per day of the canine-derived chimeric MAb cell supernatant (expressed by a CHO cell line, HI titer 1:2560, 1 mL/kg). Dog feces were collected from each group at 0–10 days after the virus challenge (Fig. 2).

The symptoms like lack of energy, depression, loss of appetite, vomiting, loose stools, sticky stools, and death were recorded daily. The amount of viral shedding of animals in each group during the treatment period was recorded. We measured new CPV-2a antigen excretion in fecal samples at 0–10 days after the challenge using the hemagglutination (HA) test and quantitative PCR (qPCR). The sequences of the primers and probe were as follows: FQ-CPV-F, 5’-CAGGAATTAACTATACTAATATATTTAATA-3’; FQ-CPV-R, 5’-AAATTT GACCATTTGGATAAACT-3’; and CPV-probe, 5’-FAM-TGGTCCTTTAACTGCATTAA ATAATGTACC-BQ1-3’.

Dogs in the challenge group were dissected immediately after death, and the tissues of the duodenum, jejunum, and ileum were collected for hematoxylin–eosin staining (HE) and immunohistochemistry (IHC) assay (He et al. 2021). A dog in the treatment group was randomly selected and used for HE and IHC assay after the 10-day experiment.

## Result

### Construction of canine-derived chimeric MAb expression vectors

**Fig. 3:**
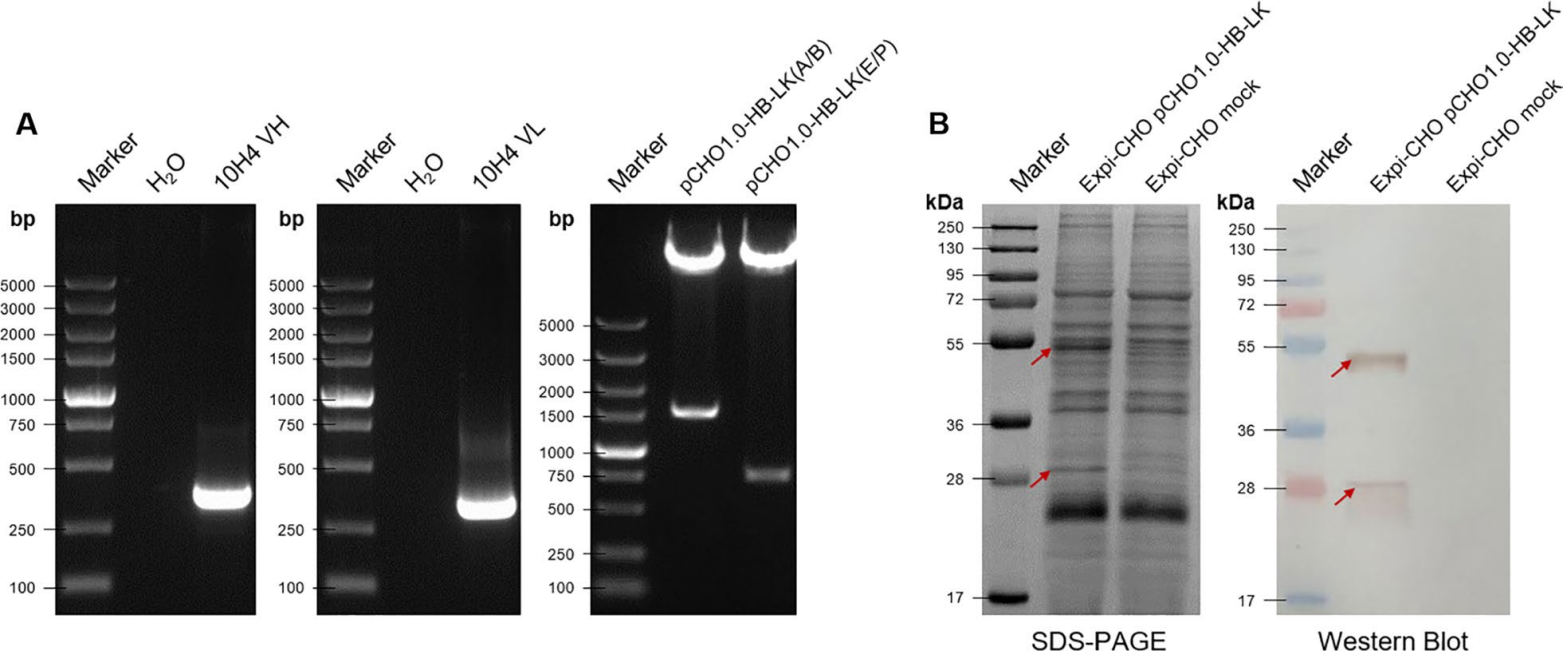
Chimeric plasmid construction and the expressed antibody identification. **A** PCR amplification of the MAb, VH (variable region of heavy chain), VL (variable region of light chain); **B** Restriction digestion of pCHO1.0-HB-Lκ, A/B (*Avr* II and *BstZ* 17I), E/P (*Eco*R V and *Pac* I); **C** SDS-PAGE and Western blot identification of the transient expression of pCHO1.0-HB-Lκ in ExpiCHO-S

The PCR fragments of 360 and 321 bp were identified as the variable region sequence of the MAb heavy chain and light chain, respectively (Fig. 3A). The constant region sequence of canine IgG class B and canine Igκ were selected. We constructed an expression plasmid (pCHO1.0-HB-Lκ) containing canine-derived chimeric IgG and chimeric Igκ (Fig. 3B).

### Transient expression of canine-derived chimeric MAbs in ExpiCHO-S cell

pCHO1.0-HB-Lκ was transfected to ExpiCHO-S cell and the target canine-derived chimeric MAbs were detected by SDS-PAGE and western blot (Fig. 3C). The HI titer of the cell supernatant in the transient expression system to new CPV-2a was 1:640, and the neutralization titer was 1:2881.8.

### Selection of stable cell lines for canine-derived chimeric MAb production

**Fig. 4:**
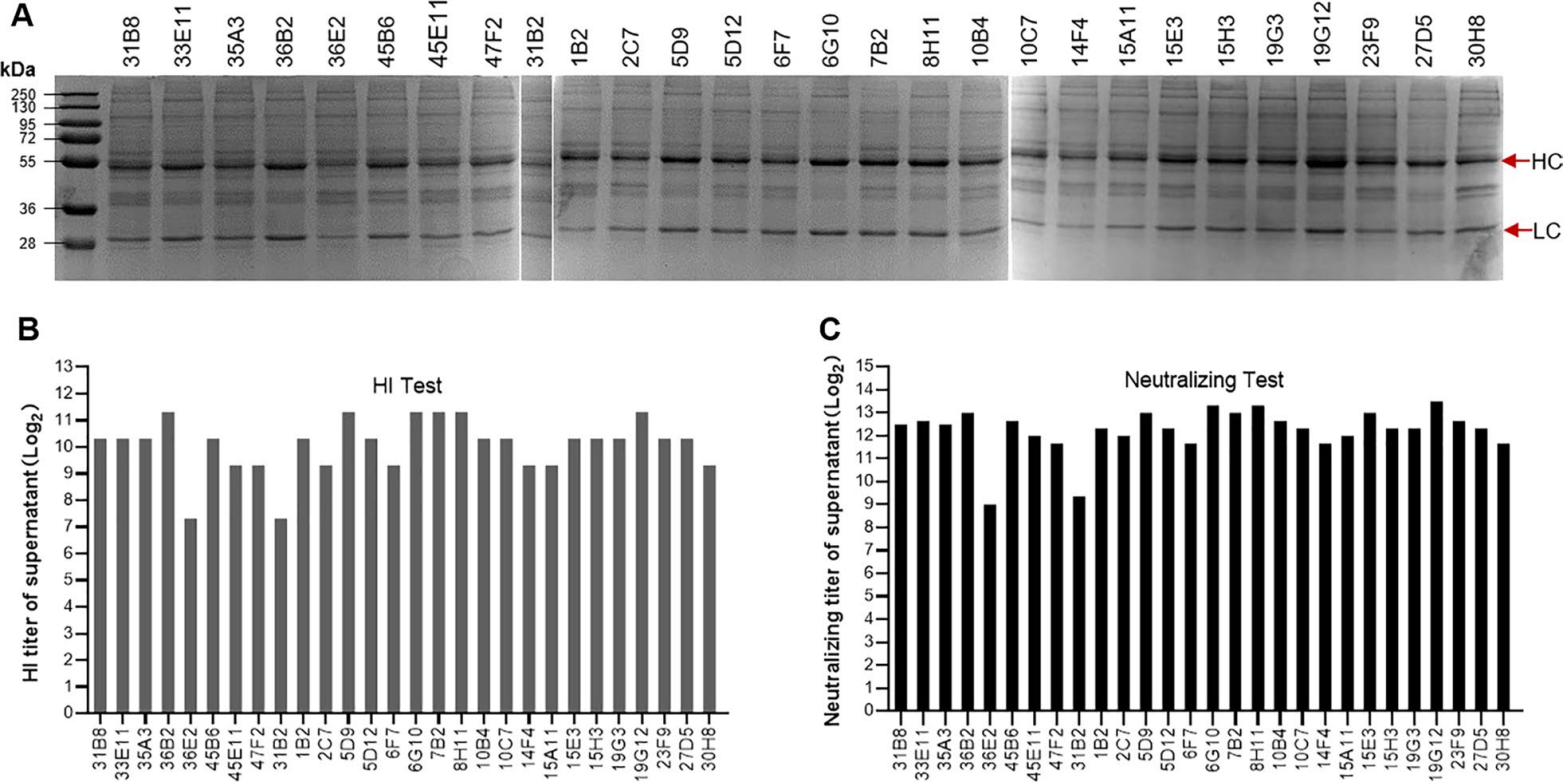
Constriction of CHO-S cell line with chimeric antibody expression: selection round 1. HC (heavy chain), LC (light chain)

After the plasmid was transferred to CHO-S cells, two rounds of pressurized screening were carried out. After limiting dilution cloning, we initially obtained 126 monoclonal cell clones, and 28 cell clones were cultivated for later evaluation after 2 rounds of selection by Dot-Blot. After 10 days of fed-batch culture, the cell supernatant from each of the 28 cell clones was evaluated by SDS-PAGE, HI, and neutralization tests, and a canine-derived chimeric MAb cell clone 19G12 was selected for further study due to the high level of protein production, and the high HI titer (1:2560) and neutralize titer (1:10,204.1) to new CPV-2a (Fig. 4).

**Fig. 5:**
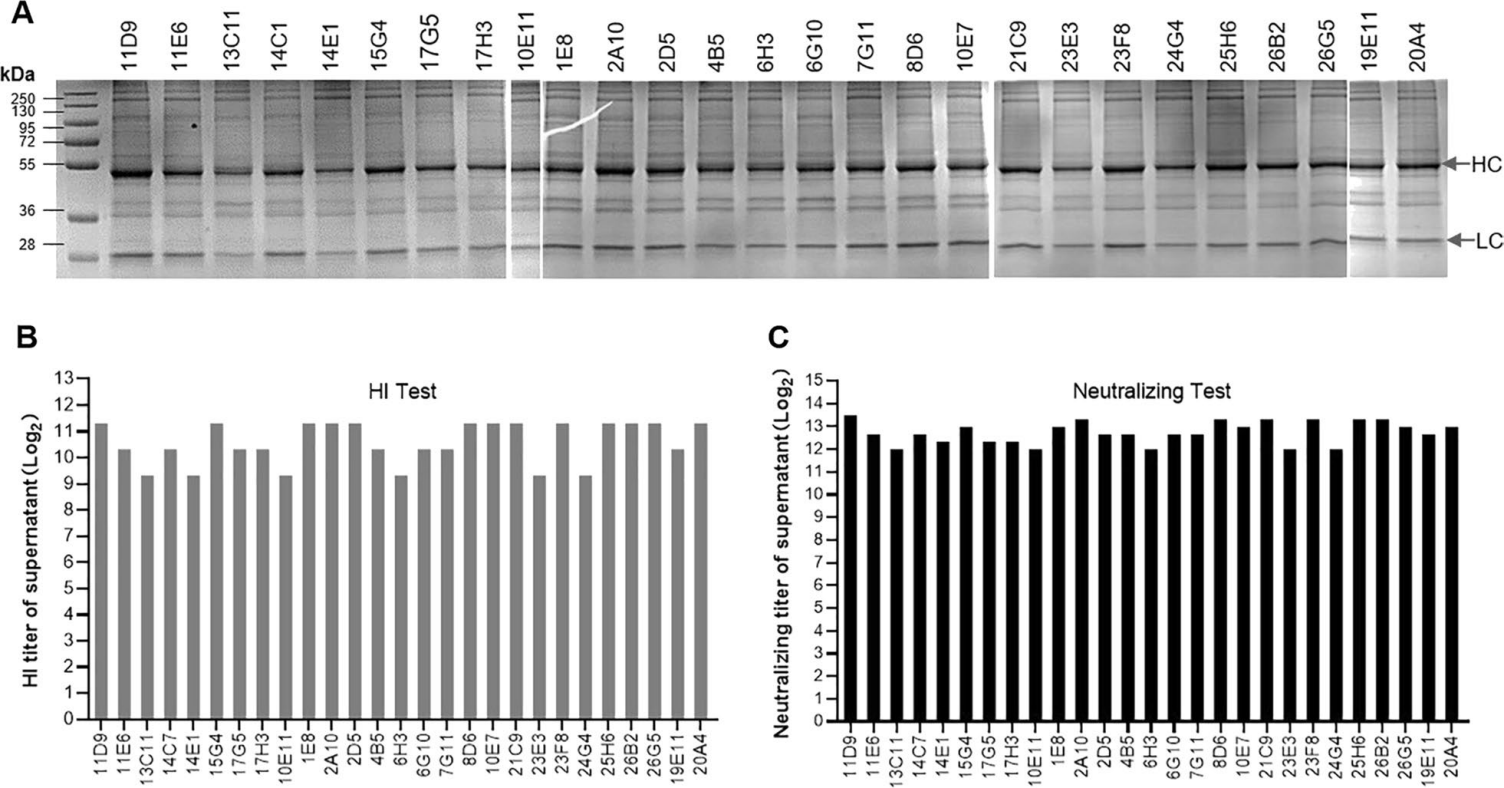
Constriction of CHO-S cell line with chimeric antibody expression: selection round 2

Subsequently, we performed the second round of limiting dilution cloning of 19G12, a total of 27 cell clones were cultivated for later evaluation after selection by Dot-Blot. After the second limiting dilution cloning, the canine-derived chimeric MAb cell line 11D9 was obtained, which showed the highest level of protein production, and the high HI titer (1:2560) and neutralize titer (1:11,494.3) to new CPV-2a (Fig. 5).

### Evaluation of the biological characteristics of canine-derived chimeric antibodies

**Fig. 6:**
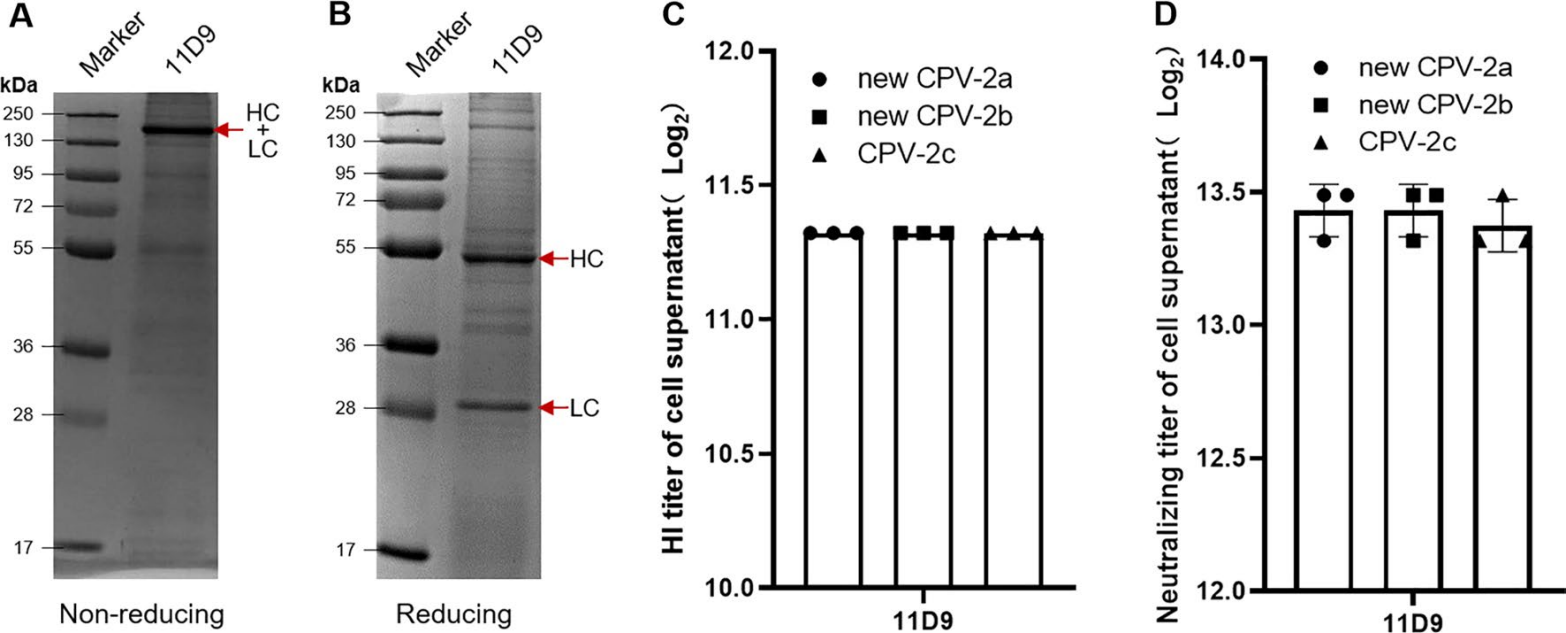
Antibody identification of 11D9. **A** Non-reducing SDS-PAGE of 11D9; **B** Reducing SDS-PAGE of 11D9; **C** and **D** The HI and neutralization titers of 11D9 to new CPV-2a, new CPV-2b, and CPV-2c

The reducing and non-reducing SDS-PAGE results showed that the light and heavy chains of canine-derived chimeric MAb 11D9 could be fully assembled (Fig. 6A and B). The HI titers of 11D9 to new CPV-2a, new CPV-2b, and CPV-2c were 1:2560, and the neutralization titers were not less than 1:10,204.1 (Fig. 6C and D).

### Therapeutic efficacy of canine-derived chimeric MAb in beagles

**Fig. 7:**
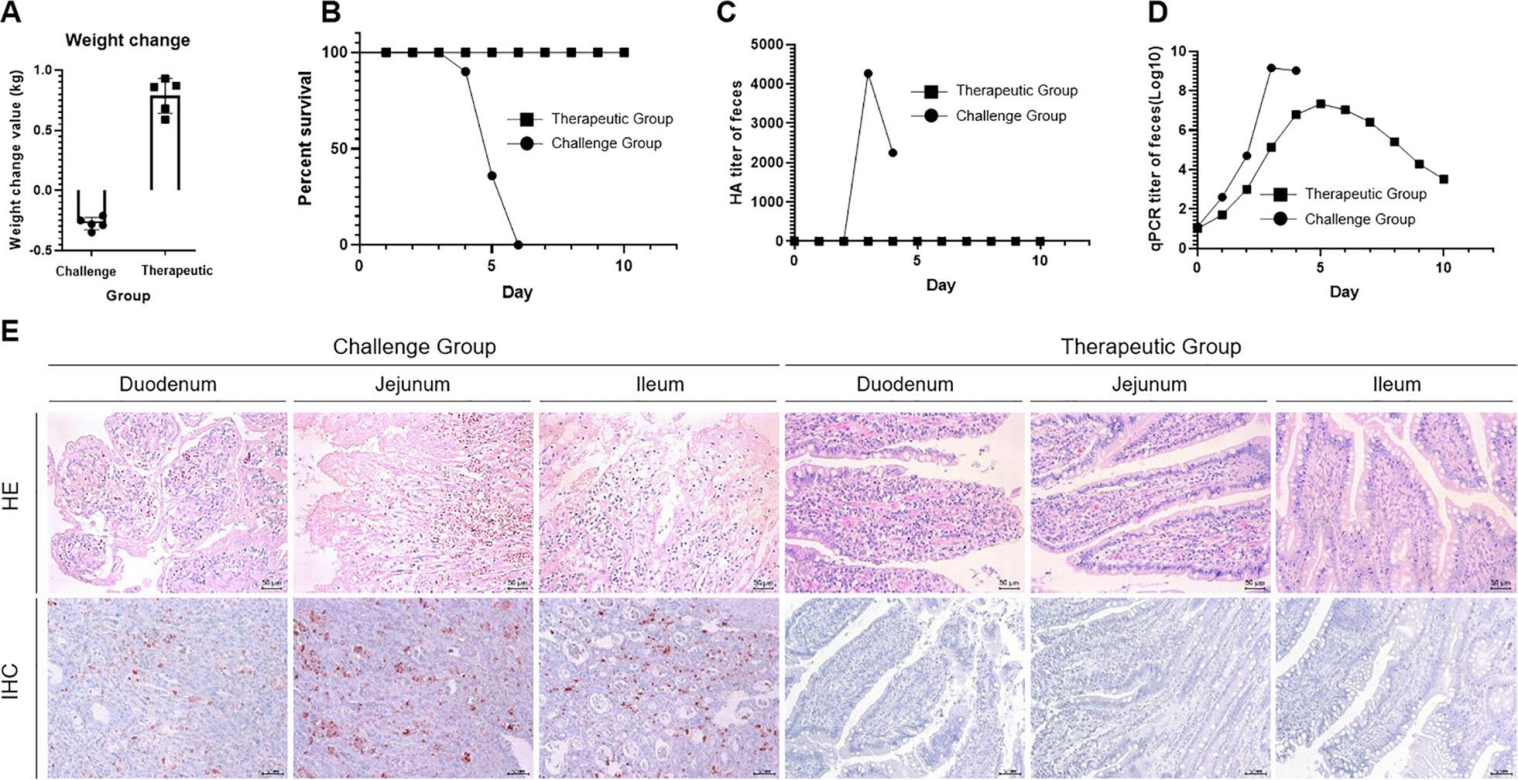
Therapeutic efficacy of 11D9 in beagles. **A** Weight changes in different groups; **B** Survival rate of different groups; **C** and **D** The HA and qPCR test of the fecal samples from different groups to new CPV-2a; **E** Pathological results of HE and IHC in the challenge and therapeutic groups

After the challenge, the dogs in the challenge group were observed to have severe symptoms of new CPV-2a infection, such as loose stools and bloody stools, depression, and loss of appetite, and all the dogs died in 6 day-post-challenge (dpc), while the treatment group showed no symptoms during the whole experiment period. The autopsy results showed that the cells of the duodenum, jejunum, and ileum in the control group exhibited massive necrosis and congestion and the number of lymphocytes decreased significantly, and dogs in the treatment group with no pathological changes. After 10 days, the weight of the dogs in the treatment group significantly increased, while the dogs in the challenge group were significantly reduced, the dogs in the challenge group died on the 4–6 dpc, while there are no symptoms observed in the dogs from the treatment group. (Fig. 7A and B).

The results of the HA test and qPCR showed the fecal viral load rose sharply from 0–3 dpc, and the fecal viral load of the treatment group continued to decrease after a slight increase (Fig. 7C and D). We collected only a small amount of blood in the stool on the 4 dpc, which may be the reason why the fecal virus content decreased on the 4 dpc.

HE results showed obvious pathological changes in the intestines of dogs in the challenge group. The intestinal epithelial cells of the duodenum were congested and swollen or atrophied, the epithelial cells of the upper intestinal villous mucosa of the jejunum were necrotic and congested, and the epithelial cells of the upper intestinal villous mucosa of the ileum had necrosis. However, there was no damage to the intestinal tissue of the dogs in the treatment group (Fig. 7E). The IHC results showed an obvious CPV-2-positive reactions in the intestinal tissue of the challenge group, while there were no positive reactions in the samples from the treatment group. These results indicated that the intestinal tissue of the dogs in the treatment group was not invaded by CPV-2 (Fig. 7E).

## Discussion

As per existing information, canine IgG antibodies are divided into four categories, A, B, C, and D, of which category B can trigger a good ADCC and Fc effect in dogs (Bergeron et al. 2014). To overcome the difficulty of accurately querying the sequence of canine Igκ in Genbank, the IMGT was used to determine the sequence of the canine light chain constant region (Ehrenmann et al. 2010).

As a canine-derived chimeric MAb, 11D9 is a genetically engineered chimeric molecule that includes the variable regions of the heavy and light chains of the mouse MAb 10H4 and canine antibody constant regions. Previous reports confirmed that human anti-murine antibodies produced by the human body are mainly directed against the constant region proteins of mouse MAb (Hosono et al. 1992). Theoretically, the immunogenicity of the canine-derived chimeric MAb 11D9 as a heterologous protein should be greatly reduced (Stephens et al. 1995). As we possess the complete gene sequence of this antibody, it would be very convenient to continue to analyze and modify it in the future.

CPV-2derived from FPV with the mutation in the 93 and 323 amino acid sites, which make it can bind to the TfR protein in dogs (Miranda and Thompson 2016). As the virulence protein of CPV-2, VP2 played an important role in the mutation and evolution of CPV-2 (Callaway et al. 2017; Parker and Parrish 1997). The difference in the VP2-specific amino acid epitopes of the virus is necessary for the virus to infect different species (Llamas-Saiz et al. 1996), and artificial changes to these sites lead to the conversion of these viruses, such as FPV and CPV-2 infecting species (Chang et al. 1992; Miranda and Thompson 2016). We used the chimeric antibody 11D9 to neutralize FPV and found that neither of them could react with FPV. Together with the antigen protein CPV-2 VP2 targeted by the MAb 11D9, these results may indicate that this antibody is directed against the specific antigenic site of CPV-2.

Neutralization tests of chimeric antibody were performed to explore its protective efficacy against mutant strains of CPV-2, new CPV-2a, new CPV-2b, and CPV-2c. The in vitro neutralization test results show that the neutralization of chimeric antibodies and murine MAb to CPV-2c was slightly weaker than that of new CPV-2a and new CPV-2b. This may be due to the relationship between new CPV-2a and new CPV-2b was better than that of CPV-2c. CPV-2 is a severely mutated DNA single-stranded virus. The classification of its variant strains mainly depends on the difference in the important amino acid positions of the VP2 protein, and the new CPV-2a and new CPV-2b have a closer relationship in comparison (Chang et al. 1992; Chen et al. 2021; Qi et al. 2020). MAb 11D9 showed slightly higher neutralizing activity against new CPV-2a than CPV-2c, which may partly be due to the sequence divergence of different CPV strains, which requires further study.

Comprehensive animal experiment results confirm that the chimeric antibody has obvious therapeutic effects. We conducted two repeated experiments to determine that the chimeric antibody can be used to treat dogs infected with new CPV-2a. However, we did not challenge animals with other types of CPV-2 variants and did not perform therapeutic trials. When combined with the neutralization potency of the previous chimeric antibody against the three variants of CPV-2, this antibody can neutralize different variants of CPV-2. What we need to explore next is how many doses per day of chimeric antibody should be injected when treating dogs infected with different CPV-2 variants.

It is worth noting that during the animal experiments, the control dog developed extremely severe symptoms and the stool collected on the 4th day was mostly bloody, so the virus content in the statistical data was reduced. The results of the dog fecal qPCR of the challenge group indicate an increased proliferation of CPV-2 after the challenge. Combined with the results of stool qPCR in the treatment group, it was determined that MAb 11D9 significantly inhibited the proliferation of CPV-2. The HA test was unable to detect CPV-2 in the treatment group, indicating that MAb 11D9 has excellent therapeutic effects.

In conclusion, the canine-derived chimeric MAb 11D9 produced by CHO-S cells showed a high HI and neutralization titer against CPV-2. Potent therapeutic effects against the new CPV-2a were observed in beagles, providing potential for the prevention or treatment of CPV-2 infections in dogs. Moreover, the successful construction of MAb 11D9 indicates an initial platform for the preparation and evaluation of other canine-derived antibodies.

## Data Availability

The datasets generated during and/or analyzed during the current study are available from the corresponding author on reasonable request.
